# The Psychological Effects of Imprisonment: The Role of Cognitive, Psychopathic and Affective Traits

**DOI:** 10.5964/ejop.3995

**Published:** 2022-08-31

**Authors:** Tiziana Lanciano, Lidia de Leonardis, Antonietta Curci

**Affiliations:** 1 Department of Education, Psychology, Communication, University of Bari ‘Aldo Moro’, Bari, Italy; California University of Pennsylvania, California, PA, USA

**Keywords:** psychological effects, imprisonment, inmates, affect, mood, anxiety, depression

## Abstract

The present cross-sectional correlational study aimed to investigate a set of cognitive, affective, and personality traits impacting the psychological effects of imprisonment. Ninety-three male inmates filled out a battery including measures of intelligence, working memory, psychopathy, aggressiveness, anxious trait, emotionality, rumination styles, and empathy proneness. Inmates' psychological outcomes were conceptualized in terms of mood, anxiety, depression, and general health. Results showed that inmates with high cognitive abilities, psychopathic impulsivity, proactive aggression, personal distress and fantasy, anxious and negative emotionality are mainly prone to ill-being psychological outcomes. Contrariwise, the fearless dominance trait, positive emotionality and empathic concern ability seem to expose inmates to positive psychological outcomes. Reactive aggression and perspective taking seem to impact both positive and negative moods. Ruminative style was unrelated to psychological outcomes. These preliminary results provide an insight into which factors intervention programs should be based upon in order to enhance well-being and reduce distress among inmates.

The present cross-sectional correlational study aimed to investigate a set of cognitive, affective, and personality traits impacting the psychological effects of imprisonment, including positive and negative moods, anxiety, depression, and general health. The study had no intention of focusing on the causes, but instead focused only on portraying the extent to which some traits may account for differential responses to imprisonment in terms of inmates’ well-being and distress. The theoretical frame supporting the current study is the product of reflection on the effectiveness and the usefulness of a prison sentence. The punishment and its implementation in prison must be as close as possible to the personality and characteristics of the offender. This would allow for the detection of risk and protection factors, as well as specific, individualized and personalized resources for each inmate (Carillo, 2007). Along with the retributive, intimidating, and defense/prevention function, punishment has a resocializing function geared toward the social recovery of the offender. Rehabilitation treatment must be individualized: It is therefore important to know some of the inmates’ characteristics (e.g. cognitive, emotional, personality skills and/or deficits) and to implement programs that include activities tailored to suit these features.

The current study is the product of an awareness that to maximize the possible positive effects of imprisonment and the effectiveness of rehabilitation programs, it is important to obtain a personalized assessment of inmates' dispositional characteristics, in terms of their skills or shortcomings. The current study placed more emphasis on the cognitive, emotional and personality factors affecting the psychological outcomes of detention than on the various effects of detention, i.e. positive/well-being vs. negative/ill-being. For example, is empowering empathic skills or cognitive abilities always a useful strategy for the improvement of well-being? Alternatively, might it be necessary to manage ruminative tendencies in order to reduce distress? Research questions such as these guided the present study. Without making sophisticated experimental claims, the present study attempts to make a contribution in this direction.

## The Psychological Effects of Imprisonment

There has been a noticeable upsurge in interest regarding the psychological and physical effects of imprisonment on inmates (Morgan et al., 2019; Picken, 2012). The medical and psychosocial relevance of these issues is constantly increasing in line with the worldwide growth in the number of incarcerated people. Investigating the psychological effects of the imprisonment is fundamental in order to reduce inmates’ pain and suffering and to prevent long-lasting consequences beyond the prison sentence itself (van Ginneken et al., 2019; Ward & Stewart, 2003). Living in prison is a traumatic and illness-risk experience: Some of the main factors related to inmates’ health during their imprisonment are traceable to overcrowding (Haney, 2012), family deprivation (Hagan & Dinovitzer, 1999), fear of the unknown, the emotional climate of distrust (Picken, 2012), and the isolation. Research into the psychological effects of solitary confinement has yielded consistent results concerning its devastating effects on mental and physical health, generally suffered by inmates in isolation. Psychological distress reaches high peaks and results in a wide range of daily adverse psychological outcomes (Arrigo & Bullock, 2008; Haney, 2018). Empirical evidence converges in considering the experience of imprisonment as being commonly characterized by high levels of stress, anxiety, low self-esteem, loneliness, and depression (Castellano & Soderstrom, 1997; Palmer & Connelly, 2005; Reitzel & Harju, 2000). Generally speaking, incarcerated people may experience a real existential crisis, by feeling anxiety and distress concerning their past and future life goals (Crewe et al., 2017).

Over the years, various approaches have been taken towards the issue of imprisonment and its effects on inmates' lives. Even if the mostly widespread conceptualizations have emphasized a common psychological deterioration following imprisonment, empirical evidence has suggested that the long-term effects of imprisonment are not the same for everyone. There are instead a number of individual differences that make some inmates resilient but which put others at greater risk of suffering distress (Picken, 2012; Tomar, 2013). A recent study regarding how inmates serving long sentences adapt to circumstances revealed that *‘most prisoners demonstrate a shift from a form of agency that is reactive to one that is productive, as they learn to “swim with”, rather than against, the tide of their situation*‘ (Crewe et al., 2017, p. 517).

Early main theories on the psychological effects of imprisonment include *'deprivation models'* and *'importation models'*. The former indicate that maladaptation and distress are ascribable to the ‘pains of imprisonment’ (Sykes, 1958), mainly due to the condition of deprivation of liberty, autonomy and security. The later models, however, suggest that mental illness is imported from an inmate's history into the prison environment (Carroll, 1974; Edwards & Potter, 2004). The limit of *'deprivation models'* is that they do not sufficiently take into account individual differences in responses to imprisonment, e.g., inmates' resilience, adjustment, and copying styles (Bonta & Gendreau, 1990; Dye, 2010). Not all prisoners experience psychological deterioration, and not all with the same intensity and modality (Fazel & Danesh, 2002). On the contrary, *'importation models'* suffer from not having properly considered the devastating psychological effects of prisons. A third approach includes *'combined models'* which integrate the psychological risk to which prisons expose inmates, and the effects of the characteristics of the inmates themselves (Liebling et al., 2005; Slotboom et al., 2011). These combined models acknowledge that the prison enviroment is highly distressing and that some prison conditions increase the likelihood of risk illness. However, these models also recognize individual differences underlying the subjective experience of imprisonment and specific vulnerabilities (Dye, 2010; Liebling et al., 2005; Yang et al., 2009), such as socio-demographic variables, cognitive resources, psychiatric history, personality, socio-emotional competences, etc. Both individual pronenesses and contextual dimensions may impact on the well-being of incarcerated individuals, as well as features of the prison environment and prison climate, and individual leanings and predispositions.

In this vein, it would therefore be plausible to investigate the psychological effects of imprisonment from a psychologically positive perspective, in terms of 'well-being'. According to Wooldredge (1999), psychological well-being is defined as *‘reflecting inmate perceptions of insecurity, stress, depression, anger, low self-esteem and loneliness felt during incarceration’* (p. 238). Well-being has been commonly conceptualized by two broad views: Subjective well-being and psychological well-being. The first refers to the hedonic facet of well-being, in terms of happiness seeking, pleasantness, life satisfaction, and a global balancing between positive and negative affectivity (Diener, 1984). The second, however, addresses the eudaimonic facet of well-being, in terms of the fulfilment of human potential, pursuing meaningful life goals and significant interpersonal relationships (Ryff, 1989; Ryff & Keyes, 1995). It is well-established that subjective well-being and psychological distress are two distinct facets of the more wide-ranging concept of mental health (Veit & Ware, 1983). Low well-being has been commonly conceptualized in terms such as depression, low self-esteem, stress, and physical symptoms. Anxiety and depressive symptoms are among the most suitable indicators of a lack of well-being. The broad-concept of well-being in prison has been traditionally studied in terms of inmates’ ability to adjust and cope with imprisonment (Brown & Ireland, 2006; Wooldredge, 1999), whereas the present study set out to review other factors rarely investigated in the prison context. We deliberately decided to not assess inmates’ life satisfaction, preferring to focus on the particular context in which their freedom, lifestyle choices and socialization are restricted and minimized.

Several studies have addressed the topic of mental health in prison with the aim of detecting individual and/or contextual factors inhancing prisoners’ well-being (Fazel et al., 2016; Haney, 2003; van Ginneken et al., 2019). On the basis of their review, Fazel et al. (2016) suggested several clinical and research recommendations to improve prison well-being including screening, monitoring and intervention programs dealing with mental disorders, suicidal risk, substance abuse, trauma-focused, gender-specific issue, and significant daytime activity (education/courses/training). Haney (2003) outlined all the psychological challenges that most inmates are forced to face in order to avoid succumbing to the traumatic prison experience, such as emotional distress (over-control, mistrust, suspicion, alienation, PTSD symptoms), interpersonal difficulties (social withdrawal, isolation, a low level of self-esteem), prison cultural co-opting and new rules of conduct. Despite this unfortunate portrayal of the incarceration experience, Haney (2003) emphasized how the post-prison adjustment process is subject to extreme interindividual variability to the point that not all prisoners (albeit a modest percentage of inmates) are irremediably psychologically damaged by it. In this vein, this study also agrees with the idea that the psychological costs of detention are highly variable and subjective. It would therefore be desirable to focus on individual characteristics to maximize the positive effects of imprisonment and minimize the negative ones. Strictly related, van Ginneken et al. (2019) sought to investigate the link between the prison climate—in terms of both prison unit-level and individual-level perceptions—and well-being, differentiated into subjective well-being and psychological distress. The authors found that most variance for well-being of incarcerated people is explained by individual—rather than group-level perception; in particular, one’s individual perception of safety, autonomy, and good peer relations were all found to be associated with higher well-being. These results are in line with the idea of the current study to focus on individual characteristics of inmates which could contribute to improving their well-being or exacerbating their illness.

Another line of research took into account inmates’ well-being by testing the efficacy of expressive arts treatment and its benefits both at the individual-level of the inmate and at the context-level of prison (e.g., Curci et al., 2021; de Leonardis, 2019; Erickson & Young, 2010; Gussak, 2004, 2006, 2009). To illustrate, Me.N.S. S.A.N.A. is a project partially involving the authors of this study which has been carried out by the Department of Education, Psychology, Communication of the University of Bari 'Aldo Moro' together with three southern Italian Penal Institutes (de Leonardis, 2019). Based on the idea of a personalized specialized treatment for inmates in a intramural environment, the project requires:

1. A neuro-psychological assessment on entering the institute (Time 0).
2. The development of an intervention program based upon assessment screening (writing workshops; support interviews; psychotherapy; self-help groups; targeted work; sports, recreational, and/or cultural activities).
3. Periodic monitoring of intervention programs.
4. Effectiveness evaluation of intervention programs (re-test, follow-up).

Additionally, in another study involving the current sample, the authors (Curci et al., 2021) adopted an intervention program based on the writing paradigm (Pennebaker, 1997; Richards et al., 2000) to evaluate features and benefits of disclosure regarding personal experiences in prison in four daily sessions. The same measures of the current study (psychological health, anxiety, depression, and negative affectivity) were collected at the pre- and post- session; ruminative depression indices were assessed after each of the four sessions. Findings supported the benefit of the adoption of writing procedures in supporting inmates’ well-being and adjustment to imprisonment (Curci et al., 2021). Furthermore, empirical evidence provided by Gussak (2004, 2006, 2009) supported the effects of art therapy in improving the mood of prison inmates, whilst Erickson and Young (2010) found that the group art therapy with incarcerated women is effective for mental health and substance abuse treatment. Assessing and monitoring inmates’ levels of well-being and any possible protective and risk factors is fundamental for the development of individualized and personalized treatment plans, to prevent future misconduct and promote social rehabilitation.

### Cognitive Abilities

The current study considered the role of individual cognitive variables which might potentially impact the psychological effects of imprisonment. Among these, working memory (WM) represents a limited capacity system predisposed to select, organize, and integrate current goal-directed intentions and relevant information (Baddeley, 2003). Due to the crucial role of WM in adaptive daily functioning, many studies have documented that individual differences in WM capacity reflect different levels of vulnerability to stress (Klein & Boals, 2001), state anxiety, depressive symptoms, ruminative coping (Stout & Rokke, 2010), and cognitive and affective facets of subjective well-being (Pe et al., 2013). In studies on individual variability related to well-being, intelligence has been theoretically and empirically hypothesized as playing a role, albeit modest (Di Fabio & Palazzeschi, 2015; Sigelman, 1981; Sternberg & Grigorenko, 2004; Wirthwein & Rost, 2011). Recent findings reported that people with low QI experienced lowest happiness (Ali et al., 2013).

### Psychopathic Personality Traits

Considering the well-established psychopathy-crime link, we introduced this factor into our investigation of psychological effects among inmates (Vaughn & Howard, 2005). The term psychopathy refers to a set of traits characterized by impulsiveness, narcissism, impaired emotional competence, heartlessness, manipulativeness, interpersonal appeal, and irresponsibility (Hare & Neumann, 2008). Psychopathy is closely linked to a wide set of antisocial or criminal acts, proactive and/or reactive violence, and recidivism (Hare et al., 2000). The dimensional conceptualisation of psychopathy explains why some psychopathic traits may also be traced in normal people and associated with adaptive and successful achievements (‘*successful psychopathy’*; Widom, 1977). This dual soul of psychopathy—*maladaptive and adaptive*—may account for the changeable pattern of associations with various outcomes, as in the specific case of well-being-related ones. For example, on the one hand, some empirical evidence suggests that people with high levels of psychopathic traits may experience high distress and poor well-being, mainly due to their impulsiveness and poor emotional intelligence and competence (Lanciano et al., 2018). Love and Holder (2014) found that psychopathy was positively related to depression and negative affect, and negatively related to life satisfaction, happiness and positive affect. On the other hand, other findings encourage the idea that some psychopathic traits may be associated with high subjective well-being. People experiencing psychopathy oriented to maximizing their personal interests, underestimating the risk and future consequences of their own decisions on others (Foulkes et al., 2014). They are completely absorbed by their own needs and convenience and this may make them happy and satisfied. Willemsen et al. (2011) found a negative association between depression levels and the interpersonal, affective and lifestyle facets of psychopathy in a sample of inmates. Furthermore, subjective well-being is profiled by high levels of sub-clinical narcissism and low levels of neuroticism (respectively, Sedikides et al., 2004; DeNeve & Cooper, 1998), two facets strongly characterizing psychopathy (Miller & Lynam, 2003; Paulhus & Williams, 2002). Recently, Durand (2018) found that whilst the impulsive anti-social trait of psychopathy was negatively correlated with higher happiness-related features, psychopathic fearless dominance was associated positively.

### Aggressiveness

Inmates have been shown to engage in a broad range of aggressive misconducts: Proactive aggression represents a type of premeditated aggression, characterized by predatory conduct with instrumental purposes. This modality of aggression requires a careful and meticulous plan of action. Contrariwise, reactive aggression represents a kind of impulsive aggression, qualified by instinctive responses to provocations or frustrations (Anderson & Kiehl, 2014; Raine et al., 2006). Pronounced aggressive tendencies go with poor well-being, negative emotionality (Laible et al., 2014), and existential distress (van Tilburg et al., 2019). Whereas anxiety and depression can be considered along with some internalizing behavior problems, aggression represents the externalizing facet, both indicators of a lack of well-being. Externalizing problems and subjective well-being were found to be inversely related in early, mid-, and late adolescence (Suldo & Huebner, 2004). In a study on the influences of depressed mood, anxiety and aggression on diurnal cortisol rhythm in post-pubertal adolescents, Van den Bergh et al. (2008) found that depressed mood and anxiety were correlated with aggressive behaviour.

### Ruminative Style

Additionally, we aimed to assess the inmates’ tendency to experience dysphoric rumination: A stable and dispositional maladaptive strategy for coping with negative mood which involves one's emotion- and symptom-focused attention (Lyubomirsky & Nolen-Hoeksema, 1993). According to the Response Styles Theory (Nolen-Hoeksema, 1991) rumination is considered as *‘repetitive and passive thinking about one’s symptoms of depression and the possible causes and consequences of these symptoms’* (Nolen-Hoeksema, 2004, p. 107). Flourishing literature on this subject has shown that rumination is associated to greater depressive symptomatology (Nolen-Hoeksema et al., 2008), distress indicators (Morrison & O’Connor, 2008a), and suicidal risk (Morrison & O’Connor, 2008b).

### Empathy Proneness

Empathy denotes the reactions people implement when observing someone else’s emotional experience (Davis, 1983). Traditionally, literature distinguishes between an emotional/affective empathy and cognitive empathy (Queirós et al., 2018). The first refers to the individual’s emotional proneness to be sensitive to and vicariously live another's emotional state, while the second refers to the individual’s cognitive ability to correctly understand and identify another's emotional state. Previous studies reported that anxiety and depression correlated positively with the empathic affective facet of personal distress, and negatively with the empathic cognitive facet of perspective taking (Grynberg et al., 2010). Other studies showed that high levels of affective empathy appeared to be associated with increased levels of depressive symptoms, while cognitive empathy was found to be separate from depressive symptoms (Gambin & Sharp, 2018).

## Aim and Hypotheses

The current study investigated the role of cognitive, personality, and affective traits in impacting the psychological effects of imprisonment. The study explored individual differences in the effects of imprisonment in order to adjust the imprisonment experience as much as possible to the individual characteristics of the inmate in terms of his/her cognitive, affectivity and personality resources and deficits. A careful and accurate assessment and monitoring of inmates' levels of well-being may prove useful to prevent risk-taking behaviors and to encourage social reintegration where possible. The relevance of the current study, as opposed to those studies mentioned above, lies in considering a range of variables rarely investigated simultaneously within the field of prison research (e.g., cognitive abilities, working memory, ruminative style, and empathy), and provides suggestions to implement specific training based on significant predictors. Additionally, inmates’ psychological outcomes were conceptualized in terms of positive and negative mood, state anxiety, depression, and general health. Based on the above-cited literature, we hypothesized that high traits of psychopathic impulsiveness, aggressiveness, anxiety, negative emotionality, depressive and brooding rumination would expose inmates to high ill-being psychological outcomes. Furthermore, high levels of cognitive abilities, fearless dominance and coldheartedness psychopathic traits, positive emotionality and empathic skills were expected to be associated to high well-being psychological outcomes.

## Method

### Sample

One hundred thirty-three inmates were recruited from three southern Italian prisons (Lecce, Bari, and Trani). The local prison authority and the Ethical Committee of the Department of Education, Psychology, Communication of the University of Bari ‘Aldo Moro’ approved the study according to the Declaration of Helsinki, and all participants provided written informed consent. The three Italian prisons were selected on the basis of their willingness to take part in the study. Twenty participants were excluded from the current study since they were female, three withdrew, and seventeen had been transferred to another prison, resulting in a final sample of ninety-three adult male inmates (*M*_age_ = 37, *SD* = 11.14, Age range = 18–70). Participants did not report a psychiatric or psychopathological history on the basis of their medical records. All participants were serving sentences for one or more crimes combined (32.3% for sex offences; 20.4% for crimes against property; 18.3% for mafia crimes; 15.1% for drug crimes; 14% for crimes against the person).^1^1The current sample of participants were also involved in another study about characteristics and benefits of disclosing personal experiences in prison through Pennebaker’s writing paradigm (Curci et al., 2021). Participants did not receive direct compensation or a reward for their participation, except the indirect benefit of spending time with operators in order to be interviewed and to answer the questionnaires.

### Measures and Procedure

The second author, a prison executive officer and a psychology PhD student with great expertise in forensic psychology, conducted or supervised all individual sessions with the help of psychologists, educators and experts from the treatment team. Participants were contacted by prison staff and invited to participate in a study concerning the emotional and cognitive processing underlying inmates’ imprisonment. In order to ensure a climate of collaboration rather than suspicion, the prisoners/participants were reassured as to the purposes of the study and the guarantee of their anonymity. Research staff provided help and support with completing the questionnaires to anyone who had difficulties with understanding but this assistence did not affect or alter the inmates’ responses in any way. Data were collected anonymously in individual sessions typically lasting around 3 hours each in rooms in prison dedicated to teaching and laboratory activities. All inmates provided written informed consent. The order of administration of the instruments making up the battery was randomised across participants. Once participants had finished they were fully debriefed and thanked individually. The only difficulty arose with some inmates who had a strong need to converse and chat. This was managed by inviting those in question to first complete the questionnaires and then to spend some time freely conversing. Data collection started in October 2017 and ended in December 2017.

#### Cognitive Abilities

##### Intelligence

Intelligence was assessed using the Italian version of Raven’s Standard Progressive Matrices (Raven, 1938).

##### Working memory

Working memory capacity was assessed with the Digit Span Forward Test (Orsini et al., 1987).

#### Personality Traits

##### Psychopathy

Participants answered the Italian version of the Psychopathic Personality Inventory-Revised (La Marca, Berto, & Rovetto, 2008; Lilienfeld & Widows, 2005). For the aim of the current study, we considered three factors: Self-Centered Impulsivity, Fearless Dominance, and Coldheartedness.

#### Affective Traits

##### Aggressiveness

The Reactive-Proactive Aggression Questionnaire (Raine et al., 2006) was administered. The two dimensions of Reactive and Proactive Aggression were considered with higher scores indicating higher levels of aggression.

##### Trait Anxiety

The participants’ trait anxiety was assessed with the Trait subscale of the State-Trait Anxiety Inventory-Y State (Spielberger, 1983). This is a self-reported inventory with the highest score indicating the greatest anxiety trait.

##### Emotionality

The participants’ emotionality was assessed with the Trait subscale of the Positive and Negative Affect Schedule (Watson, Clark, & Tellegen, 1988). Items were summed into a Positive and Negative Trait Affect score.

##### Ruminative Style

Participants’ ruminative style was assessed with the Ruminative Response Scale (Treynor et al., 2003). This measures how often people engage in ruminative responses subsequent sad or depressive moods. Items were averaged into three indices of Brooding, Depression, and Reflection.

##### Empathy Proneness

Empathy skills were assessed using the Interpersonal Reactivity Index (Davis, 1980; 1983) assessing: a) Perspective Taking (*tendency to spontaneously adopt the psychological point of view of others*), b) Fantasy (*tendency to transpose one’s self imaginatively into the feelings and actions of fictitious characters in books, movies, and plays*), c) Empathic Concern (*‘other-oriented’ feelings of sympathy and concern for unfortunate others*), d) Personal Distress (*‘self-oriented’ feelings of personal anxiety and unease in intense interpersonal settings*).

#### Psychological Outcomes

##### Mood

 The participants’ mood was assessed with the State subscale of the Positive and Negative Affect Schedule (Watson et al., 1988). Items were summed into a Positive and Negative Affect State score.

##### State Anxiety

 The participants’ state anxiety was assessed using the State subscale of the State-Trait Anxiety Inventory-Y State (Spielberger, 1983). This is a self-reported inventory with the highest score indicating the greatest anxiety state.

##### Depression

 Beck Depression Inventory-II (Beck, Steer, & Brown, 1996) assesses the severity of depressive symptoms, with the highest score corresponding to the most severe depressive symptomatology.

##### General Health

 Current general health was assessed through the short General Health Questionnaire (Goldberg, 1972; 1978). A low score is equivalent to a good general health during recent weeks.

### Results

#### Relationships Among Variables

Table 1 shows descriptive analyses for all measures considered in the study: Participants exhibited medium-high levels of cognitive abilities, psychopathic traits of impulsiveness, ruminative styles, empathy proneness, state anxiety and general ill-being; higher scores of reactive than proactive aggression (*M*_RPAQ-REA_ = 9.10, *M*_RPAQ-PRO_ = 5.87) and higher levels of positive than negative emotionality (*M*_PANAS-T PO_ = 25.80, *M*_PANAS-T NE_ = 18.14).

**Table 1:** Descriptive Statistics for All Measures

Measure	*M*	*SD*
**Cognitive Abilities**		
Intelligence	107.25	14.35
Working Memory	4.96	1.22
**Personality Traits**		
Self-Centered Impulsivity	151.36	20.93
Fearless Dominance	111.94	16.18
Coldheartedness	34.06	6.96
**Affective Traits**		
Reactive Aggression	9.10	5.37
Proactive Aggression	5.87	6.03
Trait Anxiety	49.65	6.25
Positive Emotionality	25.80	6.02
Negative Emotionality	18.14	8.45
Brooding Ruminative Style	24.90	9.60
Depressive Ruminative Style	12.49	5.25
Reflection Ruminative Style	10.42	4.73
Perspective Taking	22.52	4.76
Fantasy	20.22	4.99
Empathic Concern	21.12	3.96
Personal Distress	21.42	4.36
**Psychological Outcomes**		
Positive Mood	25.26	6.16
Negative Mood	17.70	9.12
State Anxiety	50.03	6.66
Depression	17.25	8.21
General Health	30.15	8.00

Pearson’s correlations showed the extent to which cognitive, personality, and affective traits were associated to psychological outcomes (Table 2). More specifically, positive mood appeared to be positively associated with reactive aggression, positive emotionality, perspective taking, and empathic concern. Instead, negative mood seemed to be positively associated with cognitive abilities, both types of aggression, anxiety and negative emotionality, perspective taking, and personal distress. The anxiety state appeared to be positively associated with working memory capacity, anxious trait, and emphatic fantasy. The depression scores were negatively associated with positive emotionality, and positively correlated to cognitive abilities, self-centered impulsivity, both types of aggression, anxiety and negative affective traits. Finally, general health was negatively associated with positive emotionality, and positively correlated to working memory capacity, proactive aggression, anxiety and negative emotional traits.

**Table 2:** Pearson’s Correlations

	Psychological Outcomes				
Variable	Positive Mood	Negative Mood	State Anxiety	Depression	General Health
Cognitive Abilities					
Intelligence	-.07	.26*	.18	.21*	.13
Working Memory	-.16	.50**	.27**	.28**	.34**
Personality Traits					
Self-Centered Impulsivity	.03	-.09	-.08	.21*	.14
Fearless Dominance	.15	-.04	-.09	-.02	.02
Coldheartedness	.07	.02	-.03	.07	.07
Affective Traits					
Reactive Aggression	.27**	.51**	-.03	.32**	.17
Proactive Aggression	.16	.63**	.00	.26*	.21*
Trait Anxiety	-.11	.24*	.61**	.32**	.32**
Positive Emotionality	.73**	.01	-.12	-.23*	-.24*
Negative Emotionality	.08	.85**	.15	.28**	.28**
Brooding Ruminative Style	.05	.18	-.06	.15	.05
Depressive Ruminative Style	.05	.11	-.05	.09	.02
Reflection Ruminative Style	.10	.15	-.16	.07	.02
Perspective Taking	.29**	.23*	.07	.05	-.10
Fantasy	.10	.06	.25*	.17	.20
Empathic Concern	.23*	.16	.06	.03	.04
Personal Distress	-.04	.25*	.07	.10	.04

*p < .05. **p < .001.

In spite of the numerous and scattered correlations, it clearly emerges that the inmates with high cognitive abilities, psychopathic impulsivity, proactive aggression, personal distress and fantasy, anxious trait, and negative emotionality are prone to distress. On the contrary, a positive emotionality and an empathic concern ability are associated with good psychological outcomes. Reactive aggression and perspective taking seem to affect both positive and negative mood.

#### Prediction of Psychological Outcomes

To investigate the role of cognitive, personality and affective traits in impacting the psychological effects of imprisonment, we obtained a global index of psychological outcomes through an Exploratory Factorial Analysis. The five scores of Positive mood, Negative mood, Anxiety state, Depression, and General Health were entered in the analysis. The Kaiser-Meyer-Olkin’s measure and Bartlett’s test were significant (KMO = .62, χ^2^(10) = 55.85, *p* < .001). Factors with first larger eigenvalues determine the number of factors. The first eigenvalues were 1.92, 1.12, and .76 supporting a one-factor solution. The first factor accounted for almost 38.44% of the total variance (Table 3). To run the parallel analysis (PA; Horn, 1965; Steger, 2006) we used the Monte Carlo PCA software (Watkins, 2000). The PA confirmed the one-factor solution. In the current data, only the first eigenvalue (and not the second and the third) is higher than the first eigenvalue obtained through the PA (respectively, 1.27, 1.12, and .99), suggesting that the one-factor solution is to be retained (see Table 3). On the basis of the previous results, the five outcome scores were summed up in a composite index of Psychological Outcome (α = .59, range = 124–223, *M* = 173.13, *SD* = 23.62), with high scores indicating a good psychological outcome.

**Table 3:** Exploratory Factor Analysis for the Psychological Outcomes

Psychological Outcome	*h^2^*	1^st^ factor *Well-being (PA)*
Positive Mood	.23	.48
Negative Mood^a^	.27	.52
State Anxiety^a^	.45	.67
Depression^a^	.50	.71
General Health^a^	.48	.69

*Note*. *h^2^* = item communalities. *PA* = factor loadings on the principal axis.

^a^The score was reversed before running EFA.

In order to unravel the link between dispositional traits and psychological outcomes, we statistically tested the role of cognitive, personality and affective traits in predicting the psychological effects of imprisonment. To this purpose, we ran a hierarchical multiple regression model (HMR), with the Psychological Outcome composite index as the dependent variable, and cognitive abilities at Step 1, personality traits at Step 2, affective traits at Step 3.

As shown in Table 4, at Step 1 the model is significant (*F* = 16.80, *p* < .001), with the working memory capacity as the significant predictor. At Step 2, the model is significant (*F* = 8.37, *p* < .001), but not the incremental change (*R*^2^ change = .05, *p* = .09), with working memory capacity and the psychopathic trait of fearless dominance as the significant predictors. When the affective traits scores are introduced at Step 3, the model and incremental change are significant (*F* = 8.63, p < .001; *R*^2^ change = .34, *p* < .001), with working memory capacity, fearless dominance, anxiety trait and emotionality as the predictors. Jointly considered, working memory ability and dispositional traits characterized by anxiety and negative emotionality seem to expose inmates to the risk of ill-being outcomes, whereas the psychopathic trait of fearless dominance and positive emotionality protect them by increasing their general well-being.

**Table 4:** Hierarchical Multiple Regression Model on the Composite Psychological Outcome Index

	Step 1		Step 2		Step 3	
Measure	β	*p*	β	*p*	β	*p*
Intelligence	-.06	.56	-.05	.61	.05	.53
Working Memory	-.50**	.00	-.55*	.00	-.28**	.00
Step 2						
Self-Centered Impulsivity			-.15	.13	-.12	.18
Fearless Dominance			.26**	.01	.19*	.02
Coldheartedness			-.01	.91	-.04	.56
Step 3						
Reactive Aggression					-.02	.89
Proactive Aggression					-.03	.81
Trait Anxiety					-.27**	.00
Positive Emotionality					.37**	.00
Negative Emotionality					-.37**	.00
Brooding Ruminative Style					-.28	.17
Depressive Ruminative Style					.15	.35
Reflection Ruminative Style					.11	.52
Perspective Taking					-.09	.50
Fantasy					-.13	.25
Empathic Concern					.01	.97
Personal Distress					.16	.19
*R^2^*	.28	.00	.33	.00	.67	.00
Δ *R^2^*			.05	.09	.34	.00

*p < .05. **p < .01.

### Discussion

The research domain of prison experience has a long and controversial history, with behavioral researchers however agreeing that imprisonment has devastating effects on inmates' psycho-physical well-being. The current study aimed to investigate the extent to which a set of cognitive, affective, personality variables may impact the psychological effects of imprisonment. The study investigated individual differences underlying inmates’ responses to imprisonment in order to adjust the experience of imprisonment as much as possible to the individual characteristics of the inmate (e.g., cognitive, affectivity and personality features). The results obtained partly confirmed the envisaged hypotheses, while in other cases they suggested meaningful insights. Surprisingly, both correlations and regression analyses returned a strong bond between high cognitive abilities and distress: High intelligence and high working memory capacities appeared to be associated to high anxiety, depression, negative mood and low general health. We may take a moment to consider this unexpected result. A first speculative explanation may lie in a greater availability of cognitive resources employed to recall, think, and re-think significant life events, such as the experience of imprisonment. A greater accessibility of thoughts, memories and/or intrusions might lead one to suffer more. According to network models of emotion (e.g., Bower, 1981), when the memory of an emotional episode—such as living in prison or committing a crime—is accessed, the various components of the emotional reaction (i.e., physiological, sensory, experiential) are also reactivated, and people re-experience the emotional condition (Rimé, Philippot, Boca, & Mesquita, 1992). A greater availability of cognitive resources leads to a greater reactivation of the various components of the emotional episode, and this is experienced as aversive or painful. Another possible explanation may be due to a higher metacognition and self-reflection capacity to understand and reflect on one’s ‘constrained condition’. Intelligence is traditionally related to a good quality of life, above all in terms of successful achievement and attaining social resources such as education, work, and so on. These outcomes are clearly unachievable within the prison system, so it is likely that high cognitive abilities—which are not socially deployable—translate into a greater reflexive capacity about one's own condition, leading to distress.

Concerning the psychopathic personality, our findings confirmed the ‘double sided’ nature of psychopathy, with the impulsiveness trait associated with depression, and fearless dominance predicting good psychological outcomes. Psychopathic impulsive inmates exhibited poor well-being, possibly because of their poor emotional skills to adjust and cope with the adversities of prison. Contrariwise, fearless dominance implies a stress immunity in terms of an ability to remain detached from stress or emotional arousal (Lilienfeld & Widows, 2005), and this would allow inmates to reach wellbeing-related psychological outcomes.

Proactive aggression seemed to lead to greater long-term distress, while reactive aggression appeared to affect both positive and negative mood. This partially hypothesized result might indicate that a reactive tendency to aggression plays a role on the intensity of the affective state, rather than on the valence, as if this aggressive inclination made inmates more sensitive to emotion, both positively and negatively. To support, in a study on aggression and daily emotions, Moore, Hubbard, Bookhout, and Mlawer (2019), concluded that reactive aggression was positively associated with day-to-day anger and angry reactivity to negative events, and it influenced lower levels of everyday happiness but greater happy reactivity to positive events. The authors indicated that reactive aggression is characterized by significant daily emotionality, whilst proactive aggression is characterized by a lack of emotionality.

Concerning dispositional affectivity, inmates with high negative emotionality were mainly prone to distress: Negative affectivity trait represents an emotional reactivity characterized by a general subjective distress and unpleasant feelings which include anger, dislike, repugnance, guilt, fear, and uneasiness (Watson et al., 1988). On the other hand, positive emotionality seemed to guarantee inmates good levels of well-being: A positive emotional reactivity corresponds to the intensity with which people feel enthusiastic, vigorous, and aroused. High levels of positive emotionality reflect high vigour, full attention, and enjoyable engagement (Watson et al., 1988). Traits characterized by a marked proneness to anxiety and negative emotionality impact inmates’ psychological effects: These traits reflect a facet of unpleasantness that includes a broad range of aversive affects including anxiety, nervousness, fear, guilt, and shame, which—in turn—would prevent inmates from adapting well to prison conditions. According to the tripartite model of anxiety and depression—negative affectivity, physiological hyperarousal, positive affectivity—negative affectivity is the shared component of emotional distress, while the other two components differentiate anxiety from depression: Anxiety is defined by high levels of physiological hyperarousal (Clark & Watson, 1991), whereas depression is defined by low levels of positive affectivity (anhedonia). The high state-trait associations for the indices of anxiety and emotionality are definitely due to them being two subscales of the same instrument.

Findings suggested that the different facets of affective empathy are differently associated with mood: On one hand, empathic concern is associated with positive mood, while personal distress and fantasy would bring about negative mood. Although the ability to experience and share other’s emotions has generally been considered as an adaptive socio-emotional competency (Zahn-Waxler & Radke-Yarrow, 1990), researchers introduced the idea of an ‘emotional cost of caring’ (Smith & Rose, 2011). Empathetic distress is an interpersonal construct which refers to emotional engagement in other people’s problems and the associated distressed feelings, to the point of living them as one’s own. Literature converges on considering that responding to others’ emotions with extreme levels of empathy within particular contexts—such as prison—may result in experiencing overstated responsibility for others’ suffering, also producing clinical complications (Tone & Tully, 2014). Perspective taking seemed to affect both negative and positive mood, proving to be little discriminating in explaining well-being variance, as roughly supported by findings on cognitive empathy unrelated to depressive symptoms (Gambin & Sharp, 2018).

Contrary to our hypotheses, no ruminative style has been associated with psychological outcomes, probably because the link between ruminative style and well-being is not a linear trend but is instead mediated by other intervening factors (e.g., coping style or emotional intelligence) or different imprisonment conditions (e.g., level of prison security, the type of crime or type of sentence).

The strength of the current findings is to have considered variables seldom investigated in prison research, providing suggestions to prevent risk-taking behaviors and to implement specific well-being-based training. It would seem that any training increasing the ability to 'feel other people’s emotions' or increase the metacognitive ability of memory or reflection, could intensify the inmates' emotional experience which, without adequate elaboration, might expose them to greater distress. Despite some interesting findings, the current study does, however, have some limits. First, given the correlational nature of this study, conclusions about a causal relationship cannot be drawn, mainly because it is study with data collected at a single point in time. Second, it would also be helpful to have a broader conceptualization of psychological well-being, beyond the traditional measures of psychological distress. Third, the relatively small sample and self-report instruments prevent the possibility of generalizing the results. It is likely that current results are strictly related to instruments used to assess constructs. Further studies may overcome these limitations by adopting larger samples—balanced in gender, age, nationality, crimes committed and other imprisonment-related characteristics—and a multi-traits multi-method assessment approach. Additionally, further studies may adopt a test-retest control design to test the effectiveness of intervention programs among inmates. It would be useful to investigate if and how specific dispositional traits of inmates such those here assessed (i.e., cognitive resources or emotional traits) modulate the effectiveness of prison intervention programs for well-being purpose. For example, Erickson and Young (2010) using group therapy with incarcerated women, found that art therapy is effective for participants who are defensive and have limited education and verbal skills.

Nevertheless, the results obtained, albeit modest, represent a step forward in investigating the individual differences underlying the psychological effects of imprisonment. First, a steady assessment of the dispositional characteristics considered in the current work might be a useful way to prevent high-risk cases and to identify and promote personal resources such as adaptive personality traits or positive emotionality. Each prison should have a mental illness prevention strategy including accurate assessment and monitoring of these characteristics immediately after arrival in prison and periodically throughout the entire period of incarceration. The need to evaluate inmates' abilities and traits over time in order to implement personalised treatment is strictly related to the idea that individual traits, even if relatively stable patterns of characteristics, may gradually change above all through major life events such as imprisonment (Bleidorn et al., 2019).

Moreover, the findings suggested resorting to intervention programs to promote adaptive emotional regulation strategies to best manage peaks of negative emotions (e.g., social sharing opportunities, writing paradigm activities, art workshops, etc.). Empirical evidence confirmed the health benefits of emotional disclosure in the prison context (Curci et al., 2021; Richards et al., 2000). Furthermore, one might think to improve inmates' empathic skills, but by carefully monitoring long-term outcomes, preventing possible negative aftermaths due to an excessive empathic distress. Even if preliminary, our results head in the direction suggested by the American Psychological Association guidelines, indicating the efficacy of preventive intervention programs in reducing psychological symptoms and related functioning (Hage et al., 2007). Preventive and intervention programs focus on reducing risk factors and increasing protective factors which can improve well-being and decrease distress outcomes (Kenny & Hage, 2009).
